# Multiple receptor tyrosine kinase activation related to ALK inhibitor resistance in lung cancer cells with ALK rearrangement

**DOI:** 10.18632/oncotarget.17680

**Published:** 2017-05-08

**Authors:** Se Hoon Choi, Dong Ha Kim, Yun Jung Choi, Seon Ye Kim, Jung-Eun Lee, Ki Jung Sung, Woo Sung Kim, Chang-Min Choi, Jin Kyung Rho, Jae Cheol Lee

**Affiliations:** ^1^ Department of Thoracic and Cardiovascular Surgery, Asan Medical Center, University of Ulsan, College of Medicine, Seoul, Korea; ^2^ Department of Pulmonology and Critical Care Medicine, Asan Medical Center, University of Ulsan, College of Medicine, Seoul, Korea; ^3^ Asan Institute for Life Sciences, Asan Medical Center, University of Ulsan, College of Medicine, Seoul, Korea; ^4^ Department of Oncology, Asan Medical Center, University of Ulsan, College of Medicine, Seoul, Korea; ^5^ Department of Convergence Medicine, Asan Medical Center, University of Ulsan, College of Medicine, Seoul, Korea

**Keywords:** ALK, resistance, EGFR, IGF1R, lung cancer

## Abstract

The activation of alternative receptor tyrosine kinases (RTKs) is known to mediate resistance to ALK inhibitors. However, the role of multiple RTK activation in resistance has yet to be determined. Two crizotinib-resistant (H3122/CR-1 and H3122/CR-2) and one TAE684-resistant (H2228/TR) cell lines were established. Multi-RTK arrays and Western blots were performed to detect the activation of bypass signals. There were no secondary mutations in the sequencing. EGFR and MET were activated in H3122/CR-1 cells whereas EGFR and IGF1R were activated in H3122/CR-2 cells. Concomitant activation of MET did not contribute to resistance as crizotinib completely suppressed both p-MET and p-ALK in H3122/CR-1 cells, whose survival was not affected by crizotinib. However, combined inhibition of EGFR and ALK was effective in controlling this resistant cell line. In H3122/CR-2 cells, the inhibition of both ALK and IGF1R could effectively suppress cell growth, whereas simultaneous inhibition of ALK and EGFR brought about a less-effective suppression, indicating that IGF1R activation is the main resistance mechanism. H2228/TR cells showed activation of the HER family (EGFR, ErbB2, and ErbB3). Afatinib, a pan-HER inhibitor, was more potent in suppressing resistant cells than gefitinib when combined with crizotinib, which suggests that coactivation of ErbB2 and ErbB3 also contributes to resistance. Interestingly, all three resistant cell lines responded well to AUY922, which can inhibit ALK, EGFR, and IGF1R activity. Activation of multiple RTKs can occur during acquired resistance to ALK inhibitors, in which case the dominant or significant bypass signal should be identified to provide a more appropriate combination therapy.

## INTRODUCTION

Rearrangements in the anaplastic lymphoma kinase (ALK) gene have been found in 3%–7% lung adenocarcinomas [1, 2]. ALK rearrangements are more prevalent in younger patients who have wild-type EGFR and KRAS, and in those who have never smoked or were formerly light smokers [3]. Crizotinib, an oral, selective, small-molecule ALK inhibitor, showed impressive clinical activity in patients with lung cancer associated with ALK rearrangement, leading to approval for clinical use in 2013 [4]. However, despite a rapid and dramatic initial response to the drug, the majority of patients experience disease progression commonly already within the first year of treatment [5, 6]. Similar to the T790M mutation in EGFR-TKI therapy, many secondary mutations such as L1196M, L1152R, C1156Y, and F1174L, which hinder drug binding, have been identified in crizotinib-resistant samples [5, 7].

Recently, the second-generation ALK inhibitors ceritinib and alectinib have shown promising clinical activity in ALK-positive lung cancer [8–11] and have received FDA approval for the treatment of crizotinib-refractory, ALK-rearranged lung cancer. In preclinical studies, these novel ALK inhibitors were able to overcome many crizotinib-resistant ALK mutations [7, 12, 13]. Unfortunately, resistance acquisition to these drugs is also inevitable [14, 15]. Although new agents that can cover a wider range of secondary mutations are being developed [7, 16], the limitations of these ALK inhibitors seem certain. Furthermore, there are no identifiable secondary mutations in approximately two-thirds of the cases where resistance to ALK inhibitors is observed [5, 7].

Another important resistance mechanism is the upregulation of alternate, bypass signal pathways such as those mediated by EGFR, IGF1R, and KIT [5, 7]. Among these, the EGFR pathway is the most frequently reported [5, 17]. In essence, combined treatment aiming at both the primary target and the bypass signal is necessary to overcome this type of resistance. Therefore, exact identification of the alternate pathway should be a priority. However, this can be difficult as multiple signaling pathways are often activated and they can interact with each other. Furthermore, there can be changes in the signaling pathways over time unrelated to resistance. Consequently, we cannot always be sure that the activated signal after resistance is actually responsible for this effect.

Here, we present ALK-inhibitor–resistant cell line models with activation of multiple receptor tyrosine kinases (RTKs), and discuss the significance of such activation with respect to the phenomenon of drug resistance.

## RESULTS

### EML4-ALK dependency is absent in H3122/CR and H2228/TR cells

**Figure 1:**
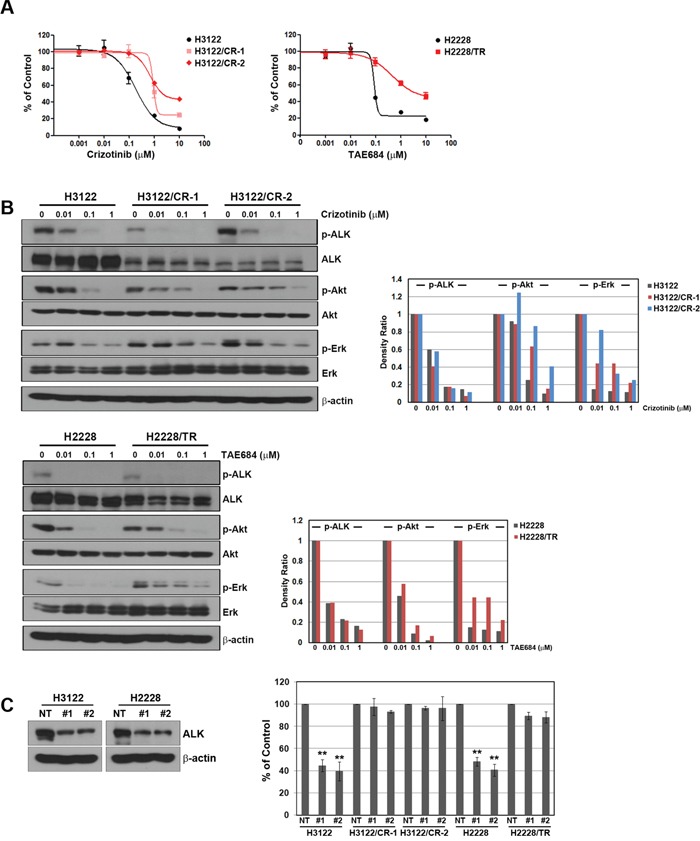
Development of acquired resistance to ALK inhibitors in the H3122 and H2228 cell lines **(A)** Cells were treated with the indicated doses of crizotinib or TAE684 for 72 h, and cell viability was then determined using the MTT assay. **(B)** Cells were treated with the indicated doses of crizotinib or TAE684 for 6 h. Molecules associated with ALK signaling activity were detected using Western blot analysis. Densitometry values were determined relative to the control after normalization to the non-phosphorylated form of each protein. **(C)** Lentiviral constructs containing the negative control (NT) and ALK shRNAs were introduced into parental or resistant cells, and ALK suppression was confirmed by Western blot analysis. Cell viability was measured by cell counting. **P < 0.001 compared with negative control shRNAs.

To identify the mechanism of action of ALK inhibitors, we generated cells that were resistant to crizotinib or TAE684. The subline with acquired resistance to crizotinib included two clones (H3122/CR-1 and H3122/CR-2) from H3122, whereas the subline with acquired resistance to TAE684 consisted of only one clone (H2228/TR) from H2228 because H2228 cells did not form colonies. As shown in Figure 1A, all resistant cells showed an approximately 10-fold higher resistance to crizotinib or TAE684 than the parental cells. Furthermore, resistant cells showed cross-resistance to other ALK inhibitors, including ceritinib and alectinib, although they showed no cross-resistance to AUY922, a HSP90 inhibitor (Table 1). Crizotinib and TAE684 could effectively inhibit ALK activation in both parental and resistant cells, but levels of phosphorylated Akt and Erk were not decreased in all resistant cells (Figure 1B). Consistent with these results, secondary mutations that can confer resistance to ALK inhibitors were not observed in all resistant cells (data not shown). To determine whether resistant cells depend on ALK signaling for growth, we suppressed the ALK gene using two different ALK-specific shRNAs, and suppression of the ALK protein was confirmed by Western blotting. Interestingly, the growth of the three resistant cell lines (H3122/CR-1, H3122/CR-2, and H2228/TR) was independent of ALK signaling (Figure 1C).

**Table 1:** Cross-resistance to other ALK inhibitors in acquired resistance to crizotinib or TAE684

	IC50 values (μM, mean ± S.D.)				
	Crizotinib	TAE684	Ceritinib	Alectinib	AUY922
**H3122**	**0.32 (±0.14)**	**0.25 (±0.11)**	**0.11 (±0.14)**	**0.08 (±1.25)**	**0.07 (±0.08)**
**H3122/CR-1**	**1.84 (±1.56)**	**1.43 (±0.75)**	**1.47 (±1.37)**	**1.84 (±0.62)**	**0.08 (±0.02)**
**H3122/CR-2**	**3.04 (±1.2)**	**2.64 (±0.53)**	**1.12 (±0.94)**	**> 10**	**0.06 (±0.04)**
**H2228**	**0.12 (±0.1)**	**0.17 (±0.24)**	**0.08 (±0.03)**	**0.14 (±0.05)**	**0.13 (±0.26)**
**H2228/TR**	**6.26 (±1.45)**	**> 10**	**3.52 (±1.02)**	**1.64 (±0.42)**	**0.25 (±0.14)**

### Acquired resistance to ALK inhibitors induces the activation of a bypass signaling

**Figure 2:**
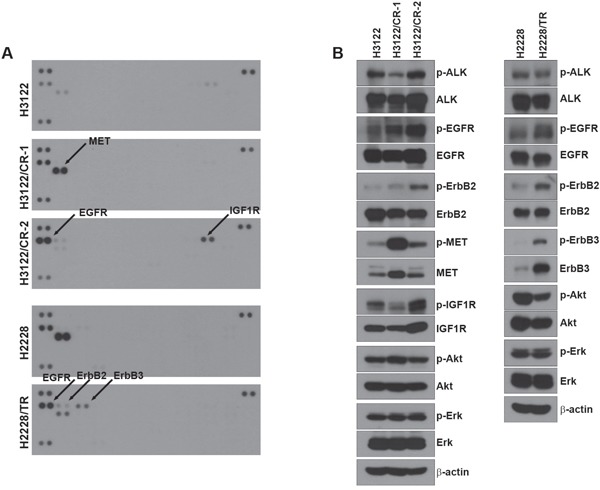
Activation of other RTKs in acquired resistance to ALK inhibitors **(A)** Cells were grown to confluence, and cell lysates were then prepared by protein extraction. 500 μg of the cell lysates were incubated on each membrane. A phospho-RTK array was performed as described in the Materials and Methods. **(B)** Lysates from each cell line were subjected to Western blot analysis. The indicated antibodies were used to evaluate the level of activated protein in Figure 2A.

We performed a multi-RTK array to identify other bypass signals contributing to drug resistance (Figure 2A). Interestingly, each resistant cell line showed activation of different receptors. H3122/CR-1 cells exhibited enhancement of EGFR as well as MET activity, whereas H3122/CR-2 cells showed activation of EGFR as well as IGF1R. In addition, the phosphorylation level of the HER family (EGFR, ErbB2, and ErbB3) was increased in H2228/TR cells compared with H2228 cells, whereas MET activity was decreased in H2228/TR cells. To confirm the results of the multi-RTK array, we performed Western blotting to evaluate the basal levels of various receptors (Figure 2B). In agreement with the data from the multi-RTK array, EGFR, MET, and IGF1R activity was found to be dramatically increased in H3122/CR cells, and HER family activation was induced in H2228/TR cells.

### Activation of EGFR, IGF1R or HER family is associated with acquired resistance to ALK inhibitors

**Figure 3:**
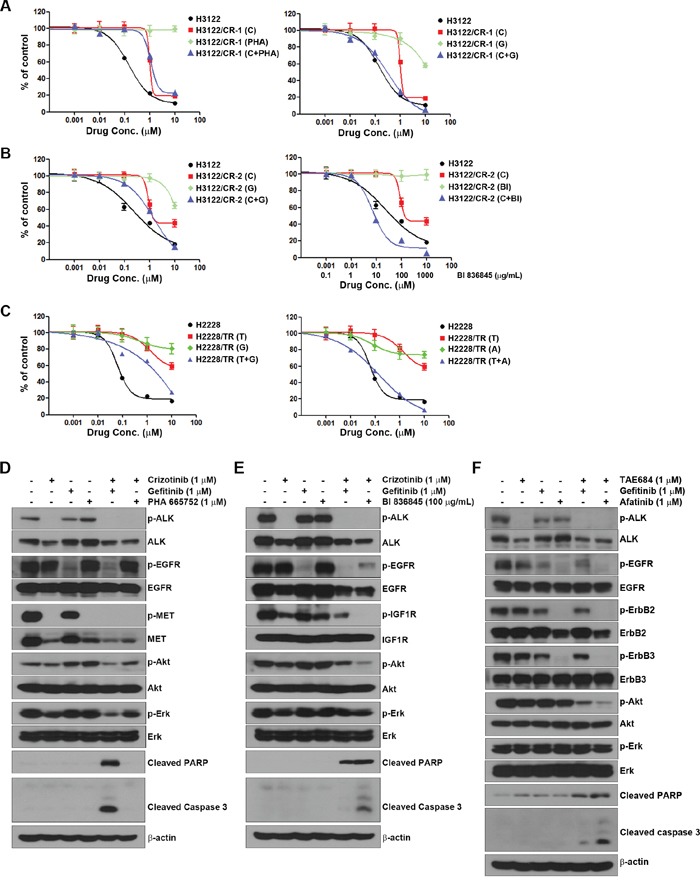
Activation of EGFR and IGF1R in acquired resistance to ALK inhibitors (**A, B**, and **C**) Cells were treated with a single drug or a combination of drugs, as indicated, for 72 h. Cell viability was measured using the MTT assay. (**D, E**, and **F**) Cells were treated with drugs as in the MTT assay. After 48 h, cells were harvested and subjected to Western blotting using the indicated antibodies. C, crizotinib; G, gefitinib; PHA, PHA 665752; BI, BI 836845; T, TAE684; A, apatinib.

To investigate whether any specific factor assessed by the multi-RTK array was associated with acquired resistance to ALK inhibitors, we used small molecules (EGFR inhibitor, gefitinib; the MET inhibitor, PHA 665752; and the HER family inhibitor, afatinib) and a ligand-neutralizing monoclonal antibody (IGF1R inhibitor, BI 836845) to inhibit each receptor. Although H3122/CR-1 cells showed concurrent activation of EGFR and MET, the combination of crizotinib with gefitinib led to restoration of sensitivity to crizotinib compared to combined treatment with crizotinib and PHA 665752 (Figure 3A). H3122/CR-2 cells exhibited activation of EGFR and IGF1R compared to parental cells. The combination of crizotinib and BI 836845 led to recovery of sensitivity to crizotinib, although combined treatment with crizotinib and gefitinib showed some additive effects (Figure 3B). Finally, H2228/TR cells showed activation of EGFR as well as the HER family. Although combined treatment with TAE684 and gefitinib showed some additive effects, this treatment only marginally restored sensitivity to TAE684, whereas resistance was completely overcome by combined treatment with TAE684 and afatinib (Figure 3C). In agreement with the MTT results, combination therapies (crizotinib plus gefitinib in H3122/CR-1 cells, crizotinib plus BI 836845 in H3122/CR-2 cells and TAE684 plus afatinib in H2228/TR cells) effectively inhibited ALK-related downstream molecules such as Akt and induced apoptosis signaling including PARP and caspase-3 activation (Figure 3D–3F).

**Figure 4:**
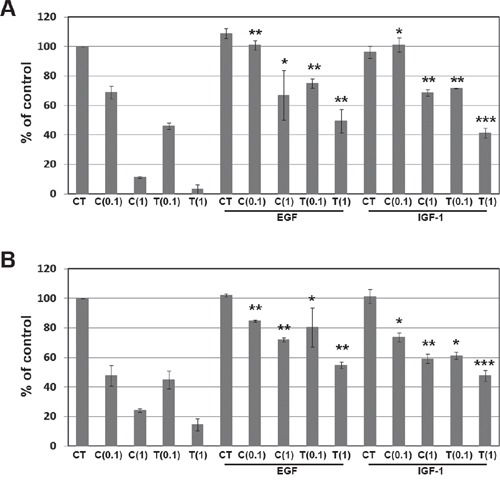
Effect of EGF and IGF-1 on resistance to ALK inhibitors H3122 **(A)** and H2228 **(B)** cells were treated with the indicated doses (μM) of crizotinib or TAE684 plus 100 ng/mL EGF or 100 ng/mL IGF-1. Cell viability was measured by cell counting. *P < 0.01, **P < 0.001, ***P < 0.0001 compared to crizotinib or TAE684 alone. CT, control; C, crizotinib; T, TAE684.

We further examined whether EGFR and IGF1R signaling pathways could induce resistance to ALK inhibitors. Cells were treated with crizotinib or TAE684 after treatment with either EGF or IGF-1. As shown in Figure 4A and 4B, each ligand treatment decreased the sensitivity to crizotinib or TAE684 in H3122 and H2228 cells. Taken together, EGFR and IGF1R signaling pathways could induce the primary as well as acquired resistance to ALK inhibitors.

### AUY922 is still effective in ALK-inhibitor–resistant cells

**Figure 5:**
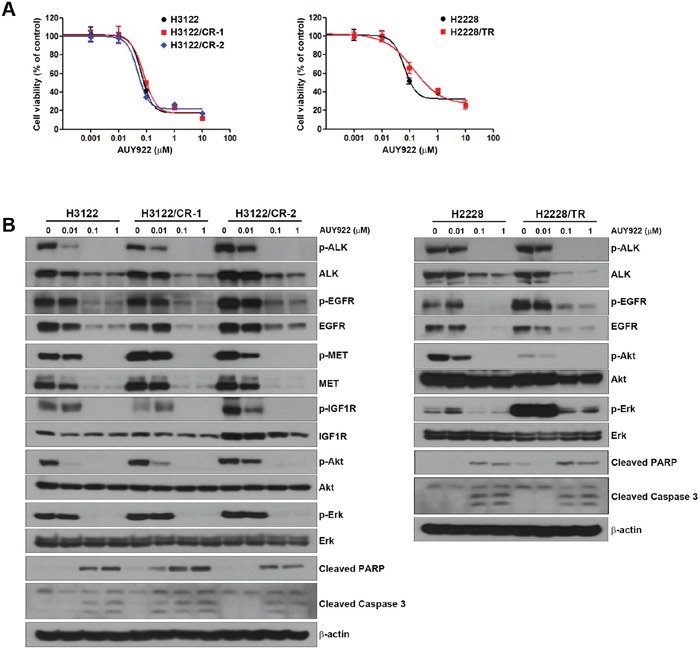
Response to an HSP90 inhibitor in H3122/CR and H2228/TR cell lines **(A)** Cells were treated with the indicated concentrations of AUY922 for 72 h. Cell viability was measured using the MTT assay. **(B)** Cells were treated with the indicated concentrations of AUY922 for 48 h. Cell lysates were subjected to Western blotting using the indicated antibodies.

Although HSP90 has a critical role in maintaining cellular protein homeostasis, it is also required for oncoprotein functioning [18–20]. In our previous studies, HSP90 inhibition resulted in EGFR, MET, and AXL degradation [21]. Interestingly, ALK-inhibitor–resistant cells showed no cross-resistance to AUY922 (Table 1 and Figure 5A). These results were similar to other Hsp90 inhibitor, 17-DMAG (Supplementary Figure 1). AUY922 treatment decreased the basal levels of ALK, EGFR, and MET, but IGF1R levels were decreased slightly (Figure 5B). This reduction led to inhibition of their activity as well as the inhibition of activity of downstream molecules such as Akt and Erk, and thereby induced apoptosis. These data demonstrate that cells with acquired resistance to ALK inhibitors remain sensitive to HSP90 inhibition through the regulation of client proteins.

## DISCUSSION

The majority of patients showing an initial good response to crizotinib experience disease progression because of acquired resistance [5, 6]. Therefore, elucidation of the resistance mechanisms, and the discovery of a strategy to overcome the problem, are required to prolong the survival in this group of patients. Several new drugs such as ceritinib, alectinib, and lorlatinib have shown efficacy in crizotinib-refractory patients [8–11]. However, these drugs with the capability to overcome many kinds of secondary mutations are still aimed at the original target, namely, ALK [12, 13]. Hence, there would be no effect in the case of activation of alternative signal pathways, and this problem can be solved by the combined inhibition of both ALK and the activated bypass signal. Unlike resistance to EGFR-TKIs where the T790M mutation is dominant, the prevalence of bypass activation in crizotinib-resistance via RTKs such as EGFR, IGF1R, c-kit, HER2/HER3, and SRC seems to be almost comparable to that of secondary mutations [22].

This study is the first to address multiple RTK activation in the context of resistance to ALK inhibitors. Some time ago we established several cell lines resistant to ALK inhibitors. In 2013, we demonstrated that the phenomenon of epithelial–mesenchymal transition (EMT) may be involved in acquired resistance to crizotinib through TGF-β1 signaling using H2228/CR cells [23]. This finding was supported by the study of Gainor et al. which detected EMT in five out of 12 ceritinib-resistant biopsy samples, although some of these had concomitant alterations [7]. During further examination of other resistant cell lines, multiple RTK activation was found in three sublines. Consistent with reports showing that EGFR signaling is the most frequently-observed alternative pathway related to resistance to ALK inhibitors, all of those resistant cell lines demonstrated EGFR activation. However, because coactivation of other RTKs was also noted in the multi-RTK arrays, clarification of its role in resistance was important.

MET and EGFR were activated in H3122/CR-1 cells. Unexpectedly, MET modulation did not affect the proliferation and survival of resistant cells, whereas combined inhibition of EGFR and ALK could overcome drug resistance. This indicates that MET activation may not be associated with resistance. We previously showed that MET activation could be induced during cancer cell proliferation, and could enhance migratory and invasive abilities without affecting cellular sensitivity to the drug [24]. Taken together with the data obtained using H3122/CR-1 cells in this study, MET activation in cancer progression might be a common phenomenon to render cancer cells more malignant, especially with respect to aspects of metastasis, without affecting the response to drugs.

In the H3122/CR-2 cell line, simultaneous increases in p-IGF1R and p-EGFR, both of which have been known to be a bypass signal related to resistance, were observed. IGF1R activation seemed to be the main resistance mechanism here, with a lesser contribution from EGFR activation. We can recognize that activation of multiple RTKs, with varying degrees of involvement, can occur during resistance acquisition. It also suggests that we should examine several candidate RTKs in tandem to identify the true agent(s) responsible for resistance. If this method of investigation is not adopted, the immunohistochemical staining of p-EGFR alone in a resistant sample could be misleading and undermine the therapeutic approach, such as in the case here.

Tanizaki et al. revealed that EGF-mediated activation of HER family signaling is associated with resistance to ALK inhibitors [25]. These authors established TAE684-resistant cell lines (TR1 and TR2) from parental H3122 cells, which demonstrated increased activation of EGFR, HER2, and HER3. The resistant cells responded well to a combination of an ALK inhibitor and an EGFR inhibitor. Our H2228/TR cell line is almost identical to those lines. When combined with the ALK inhibitor, EGFR inhibitor gefitinib also showed an anti-cancer effect; however, the pan-HER inhibitor afatinib had a more potent effect on overcoming resistance, indicating the role of HER2 and HER3 activation in resistance.

HSP90 inhibitors have shown promise with respect to reversing resistance to ALK inhibitors in preclinical models [26, 27]. However, a recent phase-II clinical trial of AUY922 in lung cancer patients with ALK rearrangements who had shown disease progression on ALK inhibitors failed to yield a positive outcome [29]. It is difficult to discern the reason for this discrepancy between preclinical and clinical studies. The drug dose tested or pharmacokinetic differences could be responsible, but even if the cause is identified this will not address toxicity issues related to use of an agent that affects such a broad range of proteins. In our study, AUY922 showed efficacy in bypassing signal-associated resistance to ALK inhibitors regardless of the type of receptor activated. As previous reports also demonstrate its efficacy regarding resistance because of secondary mutations [26, 27], HSP90 inhibitors would be ideal candidate drugs to help overcome resistance if new agents possessing clinical efficacy could be developed in the future.

In summary, activation of multiple RTKs can occur during acquired resistance to ALK inhibitors, and the dominant or significant bypass signal should be determined to provide more appropriate combination therapy. Considering the efficacy of the HSP90 inhibitor tested, its role in overcoming resistance associated with multiple RTK activation requires further exploration.

## MATERIALS AND METHODS

### Cell culture and reagents

The H2228 cell line was obtained from the American Type Culture Collection (Rockville, MD), and the H3122 cell line was a gift from Adi F. Gazdar (UT Southwestern, Dallas, TX). Cells were cultured in 10% fetal bovine serum (FBS), 100-U/mL penicillin, and 100-mg/mL streptomycin (Invitrogen, Carlsbad, CA) at 37°C in an atmosphere with 5% CO_2_. Crizotinib, TAE684, ceritinib, alectinib, gefitinib, afatinib, PHA 665752, and AUY922 were purchased from Selleck Chemicals (Houston, TX). EGF and IGF-1 were purchased from Calbiochem and Sigma–Aldrich (St. Louis, MO), respectively. BI 836845 was kindly provided by Boehringer Ingelheim (Vienna, Austria).

### Generation of ALK-inhibitor–resistant cells

H3122/CR and H2228/TR cells were established by chronic, repeated exposure to crizotinib or TAE684, as reported in previous studies [23]. H3122/CR was cloned, and these resistant sublines were designated H3122/CR-1 and H3122/CR-2, respectively. In all studies, resistant cells were cultured in a drug-free medium for >1 week to eliminate the effects of crizotinib or TAE684. The resistant cell lines were authenticated using STR analysis and confirmed to be mycoplasma free using standard methods.

### MTT assay

Cells (5 × 10^3^) were seeded in 96-well sterile plastic plates overnight and then treated with the relevant drugs. After 72 h, 15 μL of MTT solution (5 mg/mL) was added to each well and the plates were incubated for 4 h. Crystalline formazan was solubilized with 100 μL of a 10% (w/v) SDS solution for 24 h. Absorbance at 595 nm was read spectrophotometrically using a microplate reader. The results represent at least three independent experiments, and the error bars signify the standard deviation (SD) from the mean. The IC_50_ values were determined using GraphPad Prism software.

### Lentiviral infection

ALK shRNA lentiviral particles were purchased from Sigma–Aldrich. For lentiviral infection, cells were infected with the shGFP or shALK lentivirus. To validate ALK dependency, cells were infected with shGFR or shALK for 72 h, and then treated with 2-μg/mL puromycin for 24 h. Cell viability (living and dead cell number) was determined using an ADAM-MC automatic cell counter (NanoEnTek), according to the manufacturer's instructions.

### Western blotting

Whole-cell lysates were prepared using EBC lysis buffer (50-mM Tris–HCl [pH 8.0], 120-mM NaCl, 1% Triton X-100, 1-mM EDTA, 1-mM EGTA, 0.3-mM phenylmethylsulfonylfluoride, 0.2-mM sodium orthovanadate, 0.5% NP-40, and 5-U/mL aprotinin) and were then centrifuged. Proteins were separated using SDS-PAGE and transferred to PVDF membranes (Invitrogen) for Western blot analysis. Membranes were probed using antibodies against p-ALK (Tyr1604), ALK, p-Akt (Ser473), p-MET (Tyr1234/1235), p-ErbB2 (Tyr1221/1222), ErbB2, p-ErbB3 (Tyr1289), ErbB3, p-IGF1R (Tyr1135/1136), p-Erk (Thr202/Tyr204), caspase-3, PARP-1 (all from Cell Signaling Technology, Beverly, MA) Akt, Erk, p-EGFR (Tyr1173), EGFR, MET, and β-actin (all from Santa Cruz Biotechnology, Santa Cruz, CA) as the first antibody; the membranes were then treated with a horseradish peroxidase-conjugated secondary antibody. All membranes were developed using an enhanced chemiluminescence system (Thermo Scientific, Rockford, IL). Densitometric analysis was performed using the ImageJ software provided by NIH (http://rsb.info.nih.gov/nih-image/).

### Phospho-RTK array

Phospho-RTK array analysis was performed according to the manufacturer's instructions (R&D Systems, Minneapolis, MN). Experiments were performed as previously described [28].

### Statistical analysis

*P* values were determined using unpaired t-tests between comparator groups using GraphPad Prism software.

## SUPPLEMENTARY MATERIALS FIGURES AND TABLES



## Abbreviaions

RTKs: receptor tyrosine kinases
EGFR: epidermal growth factor receptor
IGF1R: insulin-like growth factor 1 receptor
ALK: anaplastic lymphoma kinase
EGFR-TKI: EGFR tyrosine kinase inhibitor
