# Supplementary material for: Multiple receptor tyrosine kinase activation related to ALK inhibitor resistance in lung cancer cells with ALK rearrangement
Source: Oncotarget. 2017 May 8;8(35):58771–80. doi: 10.18632/oncotarget.17680 (PMC5601691; doi:10.18632/oncotarget.17680)
Supplement: Supplementary file 1 [file oncotarget-08-58771-s001.pdf]

Multiple receptor tyrosine kinase activation related to ALK inhibitor resistance in lung cancer cells with ALK rearrangement

SUPPLEMENTARY FIGURE

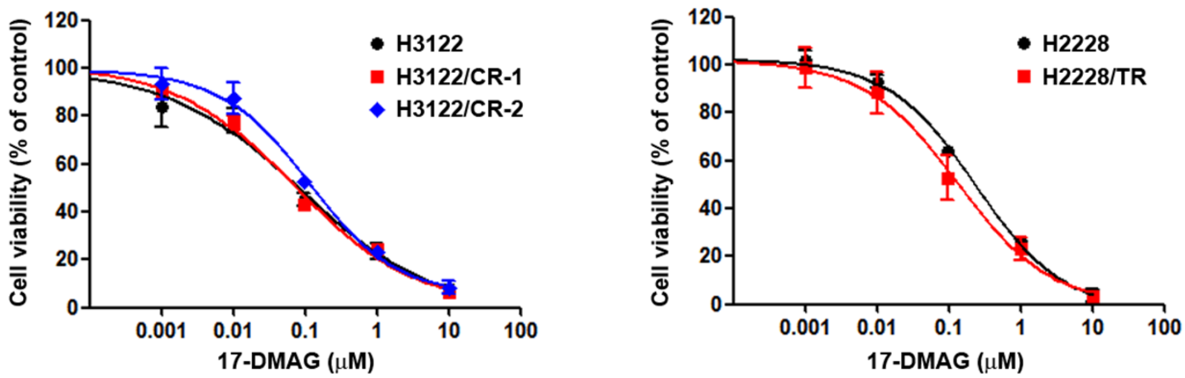

Supplementary Figure 1: Effect of 17-DMAG in cells with acquired resistance to ALK inhibitors. Cells were treated with the indicated concentrations of 17-DMAG for 72 h. Cell viability was measured using the MTT assay.
